# Necessitous Ladies' Holiday Fund

**Published:** 1916-07-08

**Authors:** Constance Beerbohm

**Affiliations:** 48 Upper Berkeley Street, London, W.


*To the Editor of* The Hospital.

### Necessitous Ladies' Holiday Fund.

*To the Editor of* The Hospital.

Sir,—For years past you have been good enough to allow me to appeal in your valuable paper for contributions towards the Necessitous Ladies' Holiday and General Fund.

Much self-sacrificing help has been given to other charities to meet the need this awful war has involved, whilst the poor ladies are likely to be overlooked, and yet no class has suffered so pitiably as the poorer gentry through loss of work, and therefore of money, more particularly hospital nurses, governesses, companions, secretaries, musicians, and actresses, and those ladies engaged in other professions who through weakness and age are disqualified for working at munitions.

I plead for these, our unfortunate sisters, the sick and broken, too proud to plead for themselves.

Any contributions sent to me to above address will be thankfully acknowledged and distributed, and I am, Sir,

Your obedient servant,

Constance Beerbohm.

48 Upper Berkeley Street, London, W., June 30, 1916.

